# Melatonin Modulates Behavioral and Physiological Responses in a Concentration-dependent Manner in a Pilocarpine-induced Seizure-like Model Using Zebrafish Larvae

**DOI:** 10.1007/s12640-026-00812-3

**Published:** 2026-07-24

**Authors:** Lorrânny Pereira de Assis Valadares, Bianca Leite Carnib, Bruno dos Santos Ferreira Roque, Felipe Cirqueira, Thiago Lopes Rocha, Mônica Rodrigues Ferreira Machado, Marina Pacheco Miguel

**Affiliations:** 1 Programa de Pós Graduação em Ciência Animal, Escola de Veterinária e Zootecnia, Universidade Federal de Goiás, Goiânia, Goiás Brazil; 2 Laboratório de Biotecnologia Ambiental e Ecotoxicologia, Centro Multiusuário de Produção e Experimentação Animal, Instituto de Patologia Tropical e Saúde Pública, Universidade Federal de Goiás, Goiânia, Goiás Brazil; 3 Laboratório de Biotecnologia e Fisiologia em Peixes, Universidade Federal de Jataí, Jataí, Goiás Brazil; 4 Setor de Patologia Geral, Instituto de Patologia Tropical e Saúde Pública, Universidade Federal de Goiás, Goiânia, Goiás Brazil

**Keywords:** Behavioral analysis, *Danio rerio*, Neuroprotection, Oxidative stress, Pilocarpine model

## Abstract

Melatonin has been extensively studied for its antioxidant activity, yet its safety profile and concentration-dependent effects remain only partially understood, particularly in aquatic experimental systems. Here, we investigated the effects of melatonin on behavioral and physiological responses in a pilocarpine (PILO)-induced seizure-like model using zebrafish (*Danio rerio*) larvae. A multibiomarker framework was employed, combining embryo–larval toxicity assessments up to 144 h post-fertilization with behavioral and biochemical endpoints related to seizure-like activity. Across the tested concentration range (0.14–18 µg/mL), melatonin did not affect mortality or hatching rates. However, exposure to higher concentrations (9.0 and 18 µg/mL) resulted in altered spontaneous contractions and increased embryonic heart rate, indicating measurable physiological changes during early development. At lower concentrations (2.25 and 4.50 µg/mL), melatonin was associated with reduced PILO-induced hyperlocomotion, increased latency to seizure-like onset, and reduced progression to more severe behavioral stages. These responses were accompanied by a reduction in reactive oxygen species (ROS) levels at both 72 and 144 hpf. Collectively, the data reveal a concentration-dependent response profile, with distinct behavioral and physiological outcomes emerging across exposure levels. This study contributes to the characterization of melatonin effects in zebrafish larvae and underscores the importance of dose selection in experimental seizure models.

## Introduction

Epilepsy is a chronic neurological disorder characterized by recurrent seizures arising from abnormal neuronal activity and continues to represent a substantial global health burden (Thurman et al. 2011). Experimental animal models have played a central role in advancing the understanding of seizure mechanisms and in the identification of candidate antiseizure compounds. Among alternative vertebrate models, zebrafish (*Danio rerio*) has gained increasing relevance in epilepsy research owing to its rapid development, high reproductive capacity, conserved neurochemical pathways, and suitability for high-throughput behavioral and toxicological analyses (Kaleuff et al. 2014; D’Amora et al. 2023; Sanz et al. 2023; Vashishat et al. 2024). In addition, zebrafish larvae enable the simultaneous investigation of behavioral, physiological, and developmental responses in an intact organism, providing a valuable platform for translational neuropharmacological studies (D’Amora et al. 2023).

Among seizure induction protocols, pentylenetetrazol (PTZ) remains the most extensively employed chemoconvulsant in zebrafish models because it produces robust and reproducible seizure-like phenotypes with well-established behavioral characterization (Baraban et al. 2005; Gawel et al. 2020). Nevertheless, PTZ-induced seizures are predominantly linked to GABAergic inhibition blockade and may not adequately reproduce mechanisms associated with cholinergic hyperexcitation. Pilocarpine (PILO), a muscarinic cholinergic agonist, offers an alternative experimental strategy capable of engaging distinct neuronal pathways involved in seizure generation (Pappano 2018; Szep et al. 2023). In rodent models, pilocarpine-induced seizures have been widely used to investigate temporal lobe epilepsy and epileptogenesis, reproducing several behavioral and neuropathological features observed in human epilepsy (Lima et al. 2006).

Although cholinergic seizure induction has also been reported in zebrafish, studies using PILO remain considerably less explored than PTZ-based approaches (Khotimah et al. 2024; D’Amora et al. 2023). Available evidence indicates that PILO exposure in zebrafish larvae induces characteristic seizure-related responses, including hyperlocomotion, tremors, tail flicking, and impaired postural control (Vermoesen et al. 2011; Gawel et al. 2024). However, investigations integrating seizure-associated behavioral alterations with developmental toxicity endpoints in this model are still limited.

Melatonin is an endogenous indoleamine widely recognized for its role in circadian rhythm regulation and antioxidant defense mechanisms (Zhdanova et al. 2001; Reiter et al. 2017; Khan et al. 2020). Beyond its chronobiological functions, growing evidence suggests that melatonin may exert neuroprotective effects in experimental epilepsy models by reducing seizure severity, oxidative imbalance, and neuronal injury (Barateli et al. 2014; Farias et al. 2022). In zebrafish, melatonin pretreatment attenuated kainic acid-induced seizure responses and oxidative stress markers, reinforcing its potential relevance in seizure modulation (Farias et al. 2022). Despite these findings, the literature remains inconclusive regarding its concentration-dependent effects. Protective responses are frequently reported at lower concentrations, whereas higher exposures have been associated with physiological and developmental disturbances, suggesting that melatonin effects may vary according to exposure level (Nnadi et al. 2025).

Although behavioral assays are frequently performed at 120 hpf because zebrafish larvae are fully hatched and exhibit stable locomotor activity, seizure-like responses can already be reliably induced at earlier developmental stages. At 72 hpf, the central nervous system is functionally established, spontaneous swimming behavior is present, and larvae exhibit reproducible behavioral responses following exposure to chemoconvulsants such as pilocarpine and pentylenetetrazol (Baraban et al. 2005; Vermoesen et al. 2011; Basnet et al. 2019). Therefore, comparing larvae at 72 and 144 hpf provides an opportunity to investigate whether developmental maturation influences behavioral susceptibility and oxidative responses during seizure-like conditions.

Despite increasing interest in melatonin as a neuroprotective candidate, its dose-dependent effects in zebrafish models based on cholinergic seizure induction remain poorly characterized. In addition, studies simultaneously addressing anticonvulsant-related responses and developmental toxicity endpoints in PILO-exposed zebrafish larvae are still scarce. Therefore, the present study aimed to evaluate the toxicity and antiepileptic effects of melatonin in a pilocarpine-induced seizure model using zebrafish (D. rerio) larvae at two distinct developmental stages (72 and 144 hpf). We hypothesized that melatonin exerts concentration-dependent neuroprotective and anticonvulsant effects, while higher concentrations may lead to measurable neurodevelopmental toxicity.

## Methodology

### Ethical Statement and Zebrafish Rearing

All experiments were performed at the Fish Biotechnology and Physiology Laboratory (LABFISH/IB/UFJ) and at the Environmental Biotechnology and Ecotoxicology Laboratory (Labae/CMPEA/IPTSP/UFG). Experimental procedures were approved by the respective Animal Ethics Committees (CEUA/UFJ, protocol No. 001/2022; CEUA/UFG, protocol No. 003/2024) and conducted in accordance with national and international guidelines for the use of animals in research.

Adult *Danio rerio* were maintained in a recirculating aquarium system under controlled conditions (27.5 ± 1 °C; 14 h light/10 h dark photoperiod). Breeders received four daily feedings, consisting of two feedings with commercial ornamental fish food (Poytara^®^) and two with live food (*Artemia salina* nauplii). All animal handling procedures followed the guidelines of the National Council for Animal Experimentation and international standards for the care and use of animals in scientific research.

### Experimental Design

To determine safe concentrations of melatonin for subsequent testing in embryos and larvae subjected to pilocarpine-induced seizures, a zebrafish embryo–larval toxicity test (ZELT) was initially performed according to OECD guideline No. 236: *Fish Embryo Acute Toxicity Test* (OECD 2013) and Lammer et al. (2009).

Embryos were obtained from 15 adult zebrafish maintained under controlled conditions using standard breeding tanks (TECNIPLAST) at a 2:1 male-to-female ratio. Viable embryos at approximately 2 h post-fertilization (hpf) were selected according to established morphological criteria described by Kimmel et al. (1995) and Valadares et al. (2023).

Embryos were randomly distributed into experimental groups and allocated into 24-well plates (five embryos per well; 20 embryos per replicate) containing 2 mL of E3 medium or test solutions. Randomization was performed immediately after embryo selection to minimize selection bias.

The experimental groups consisted of melatonin diluted in 0.1% DMSO at eight concentrations (18, 9, 4.5, 2.25, 1.12, 0.56, 0.28, and 0.14 µg/mL); negative control (E3); positive control (E3 + 4 mg/L dichloroaniline); and solvent control (E3 + 0.1% DMSO).

The ZELT was conducted over 144 h post-exposure (hpe), under static conditions, in triplicate, with controlled temperature (27 ± 1 °C) and photoperiod (14 h light/10 h dark), using a biochemical oxygen demand incubator (SOLAB SL-224/120, Brazil). Each experimental group consisted of three independent biological replicates with 20 embryos per replicate, totaling 60 embryos per group.

### Embryo Selection and Exclusion Criteria

Only viable embryos exhibiting normal morphology, intact chorion, regular cleavage pattern, and absence of developmental abnormalities at 2 hpf were included in the experiments, according to Kimmel et al. (1995).

Embryos presenting coagulation, developmental delay prior to treatment allocation, absence of fertilization, or visible structural abnormalities were excluded before randomization. During behavioral analyses, larvae presenting tracking artifacts, incomplete detection by the software, or prolonged immobility unrelated to seizure-like behavior were excluded from the final analysis dataset.

### A Multibiomarker Assessment

During the 144 h exposure period, embryos and larvae were evaluated daily for viability and teratogenicity. Embryos showing coagulation, absence of somite formation, non-detachment of the tail, or absence of heartbeat were considered dead (OECD 2013).

Teratogenic parameters assessed included embryo coagulation, lack of somite formation, failure of caudal bud separation from the yolk sac, absence of cardiac activity, and presence of edema (OECD 2013; Lammer et al. 2009; Valadares et al. 2023).

Neurotoxicity was evaluated at 24 hpe by quantifying spontaneous contractions (movements per minute) during a 60-second interval using a stereomicroscope (Leica) and a manual counter (Krzykwa et al. 2018). Cardiotoxicity was assessed at 48 hpe by counting heartbeats over 60 s to determine heart rate (beats per minute) (Babic et al. 2017). Hatching rate was monitored up to 96 hpe, considering larvae fully emerged from the chorion as hatched.

### Anti-Epileptic Seizure Assessment

Embryos at 2 hpf were selected according to Kimmel et al. (1995) and Valadares et al. (2023) and divided into two experimental induction time points: 72 and 144 hpf. At both time points, larvae were allocated into six groups: negative control (E3); pilocarpine 15 mM (PILO); PILO + melatonin (2.25–4.5 µg/mL); solvent control (0.1% DMSO); and solvent + PILO (0.1% DMSO + 15 mM PILO). Larvae were randomly distributed among experimental groups to minimize allocation bias.

A total of 15 larvae per experimental group was used for behavioral assessment. Sample size was defined based on previous studies employing zebrafish seizure-like behavioral models under similar experimental conditions (Baraban et al. 2005; Gawel et al. 2020; Valadares et al. 2023). Quality-control procedures were applied before data analysis. Exclusion criteria included impaired tracking due to overlapping larvae, image acquisition failures, and software-generated tracking inconsistencies. No larvae were excluded based on expected experimental outcomes.

Larvae were evaluated at 72 and 144 hpf because these developmental stages represent distinct phases of neural maturation. At 72 hpf, the zebrafish central nervous system is already functionally established, exhibiting coordinated swimming behavior, sensory processing, and reproducible seizure-like responses following chemoconvulsant exposure. By 144 hpf, further maturation of neuronal circuitry, neurotransmitter systems, and endogenous antioxidant defenses has occurred. Comparing these developmental stages therefore allows assessment of whether neural maturation influences behavioral susceptibility and oxidative responses to melatonin under pilocarpine-induced seizure conditions (Kimmel et al. 1995; Baraban et al. 2005; Vermoesen et al. 2011; Basnet et al. 2019).To ensure effective melatonin uptake, larvae were pretreated for 24 h prior to PILO exposure. Neurobehavioral and biochemical analyses were conducted immediately after PILO induction. Larvae designated for ROS analysis were not subjected to behavioral testing and were processed immediately after seizure induction.

### Monitoring and Scoring of Larval Behavior Post-Induction

Seizure induction was performed for five minutes, during which behavioral alterations were monitored, as described by Baraban et al. (2005). Following induction, plates were transferred to a recording chamber with bottom illumination, and ten-minute videos were recorded using a Logitech C920 camera positioned above the plate (Hong and Zha 2019).

Swimming behavior was analyzed using Matlab-based motion tracking software (Zebtrack). Individual larvae were tracked within predefined arenas corresponding to each well (internal diameter approximately 2.5 mm). Detection parameters were optimized for each video, and wells with tracking artifacts were excluded from analysis.

Video analysis was conducted by an investigator blinded to the experimental groups to reduce observer bias. Behavioral output was expressed as total distance traveled. Latency to seizure stages was assessed using BORIS software and defined as the time elapsed from the beginning of recording until the larva reached each seizure stage, as described in Table 1 (Baraban et al. 2005).

**Table 1:** Classification of seizure-like behavior

Score	Behavioral phenotype
1	Dramatic increase in swimming activity
2	Whirlpool swimming behavior and stage
3	Clonus-like seizures followed by loss of posture, when the animal falls to one side and remains immobile for 1 to 3 s

Adapted from: Baraban et al. 2005

### Evaluation of ROS Levels in an Epileptic Model

Reactive oxygen species (ROS) levels were assessed using the fluorescent probe 2′,7′-dichlorodihydrofluorescein diacetate (DCFH-DA), following Mugoni et al. (2014). Ten larvae per replicate were used, with three independent replicates per group, totaling 30 larvae per experimental group. Larvae were randomly distributed among experimental groups and replicates to minimize allocation bias. Larvae were treated with melatonin (2.25–4.5 µg/mL), 0.1% DMSO, and/or induced with 15 mM pilocarpine. Negative controls consisted of larvae maintained in E3 medium, while positive controls were larvae exposed to PILO alone. Sample size was defined according to previous zebrafish larval studies evaluating oxidative stress biomarkers under similar experimental conditions.

After treatment at either 72 or 144 hpf, larvae were washed twice with PBS (pH 7.1), homogenized in 500 µL of cold PBS using a tissue micro-homogenizer, and centrifuged at 12,000 rpm for 30 min at 4 °C. Supernatants (20 µL) were transferred to 96-well plates in triplicate, followed by the addition of PBS and DCFH-DA (10 mg/mL). Plates were incubated for 30 min in the dark, and fluorescence was measured using a plate reader (SpectraMax Paradigm^®^, Molecular Devices) at excitation/emission wavelengths of 485/530 nm. Total protein content was determined using the Bradford (1976) method. ROS levels were expressed as relative fluorescence units normalized to protein content.

Quality-control procedures were applied before biochemical analysis. Exclusion criteria included insufficient sample homogenization, technical failures during fluorescence acquisition, and samples presenting evident contamination or inconsistent protein quantification. No samples were excluded based on expected experimental outcomes.

### Statistical Analysis

Data distribution was assessed using the Shapiro–Wilk test, and homogeneity of variances was evaluated using Levene’s test prior to parametric analyses. Behavioral and biochemical data involving two independent variables (melatonin dose and developmental stage/time point: 72 and 144 hpf) were analyzed using two-way ANOVA followed by Bonferroni’s post hoc test, when appropriate. Variables involving a single experimental factor were analyzed using one-way ANOVA followed by Tukey’s multiple comparisons test. Non-parametric data were analyzed using the Kruskal–Wallis test followed by Dunn’s multiple comparisons test. No outliers were removed from the datasets. The choice of statistical test was based on data distribution and variance homogeneity. A significance level of *p* < 0.05 was adopted. Statistical analyses and graphical representations were performed using GraphPad Prism 8.0.1 (GraphPad Software Inc., San Diego, CA, USA).

## Results

### Mortality and Hatching Rates

**Fig. 1:**
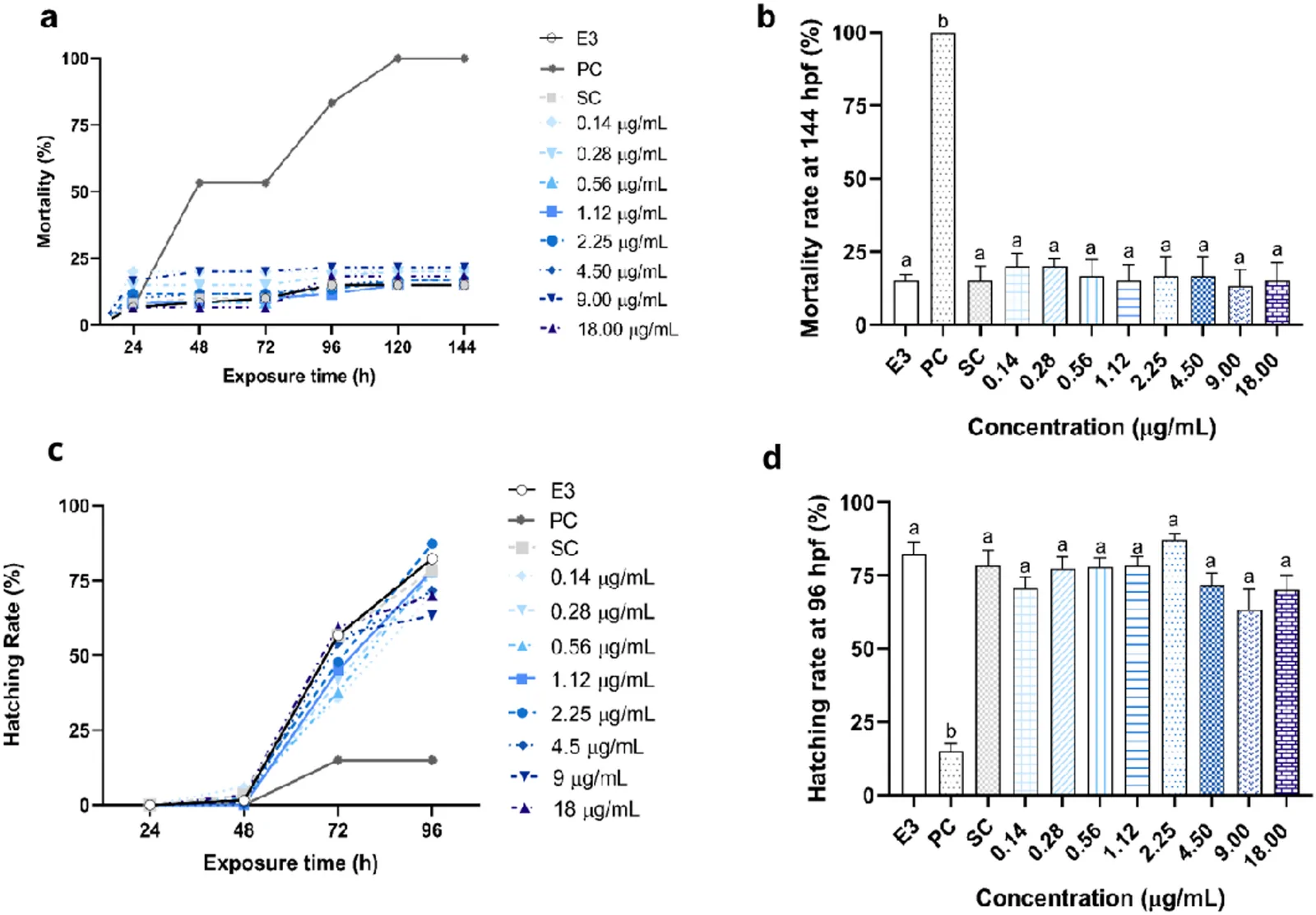
(**a**) Daily mortality rates (%) from 24 to 144 hpf of *D. rerio* embryos/larvae exposed to melatonin (18, 9.0, 4.5, 2.25, 1.12, 0.56, 0.28, and 0.14 µg/mL), including negative control (E3 medium), solvent control (0.1% DMSO), and positive control (PC; E3 + 4 mg/mL dichloroaniline) used for assay validation; (**b**) Final mortality rate (%) at 144 hpf; (**c**) Hatching rates (%) of *D. rerio* embryos/larvae exposed to different concentrations of melatonin from 24 to 96 hpf; (**d**) Final hatching rate (%) at 96 hpf. Mortality and hatching were independently evaluated, and non-hatched embryos were not considered dead unless established morphological criteria for lethality were observed. Data are presented as mean ± SD. Different letters indicate statistically significant differences between groups (*p* < 0.05). Larvae analyzed at different developmental time points belonged to independent biological cohorts. Panels “a” and “c” represent temporal progression analyses, whereas panels “b” and “d” represent endpoint measurements obtained at the final experimental time points. Temporal progression data (panels “a” and “c”) were analyzed using two-way ANOVA followed by Tukey’s post hoc test, whereas endpoint data (panels “b” and “d”) were analyzed using the Kruskal–Wallis test followed by Dunn’s post hoc test

Melatonin exposure (0.14–18 µg/mL) did not significantly alter embryo–larval mortality or hatching rates compared with the negative control (*p* > 0.05; Fig. 1a–d). Mortality and hatching data obtained across developmental time points were analyzed considering treatment and exposure time as independent variables.

As expected, the positive control (4 mg/mL dichloroaniline), included exclusively to validate assay sensitivity according to zebrafish embryo toxicity guidelines, induced complete mortality and significantly reduced hatching rates relative to the experimental groups (*p* < 0.05; Fig. 1a, b). Embryos that failed to hatch were not automatically classified as dead, and mortality was determined based on established morphological and physiological criteria, including coagulation, absence of heartbeat, and impaired somite formation.

Hatching dynamics in melatonin-treated groups remained comparable to those observed in the control groups throughout the experimental period (Fig. 1c, d).

### Neurotoxicity and Cardiotoxicity

**Fig. 2:**
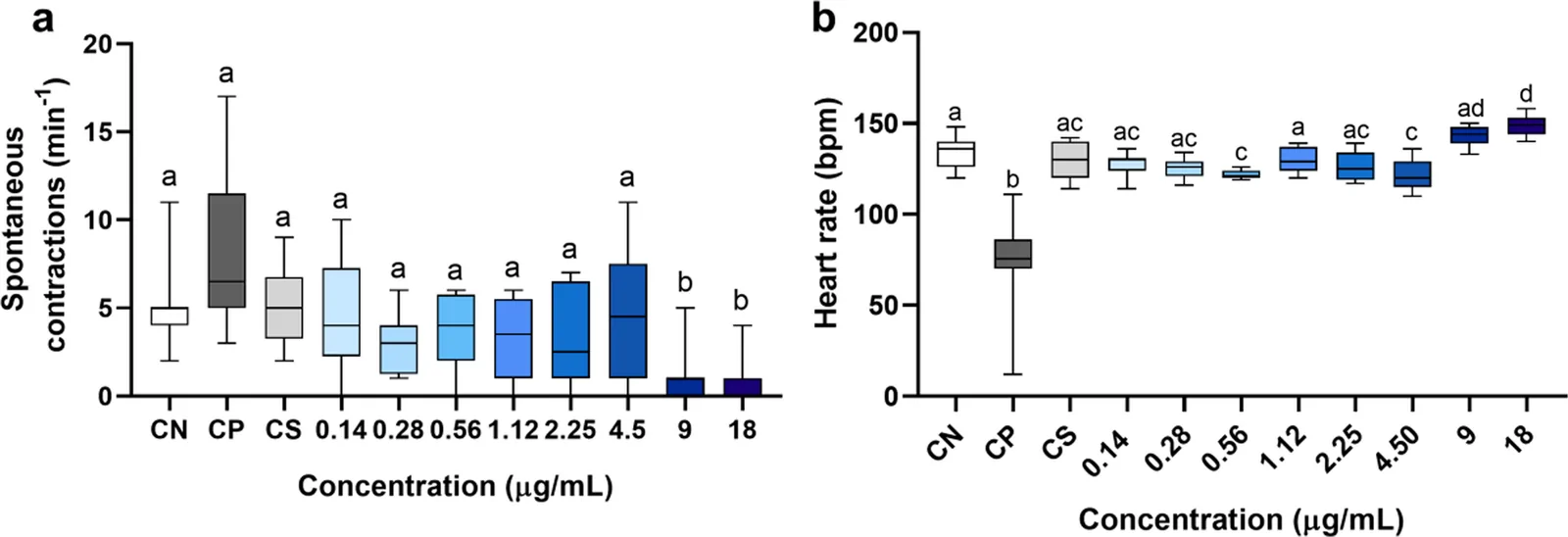
(**a**) Spontaneous contraction frequency of *D. rerio* embryos/larvae exposed to melatonin (18, 9.0, 4.5, 2.25, 1.12, 0.56, 0.28, and 0.14 µg/mL), including negative control (E3 medium), solvent control (SC; 0.1% DMSO), and positive control (PC; E3 + 4 mg/mL dichloroaniline). Evaluations were performed at 24 h post-exposure (hpe); (**b**) Heart rate of *D. rerio* embryos/larvae exposed to the same experimental conditions. Evaluations were performed at 48 hpe. Data are presented as mean ± SD. Thirty larvae per group were analyzed, with each larva considered one independent experimental unit (*n* = 30). Different letters indicate statistically significant differences between groups (*p* < 0.05). Data were analyzed using the Kruskal–Wallis test followed by Dunn’s post hoc test

At 24 hpe, exposure to higher melatonin concentrations (9.0 and 18 µg/mL) led to a significant reduction in spontaneous contraction frequency relative to the negative control (*p* < 0.05; Fig. 2a), suggesting interference with early neuromotor activity. Lower concentrations did not significantly alter this parameter (*p* > 0.05). In contrast, the positive control group exhibited increased spontaneous activity (*p* < 0.05), consistent with a stimulatory effect on embryonic movement.

Regarding cardiac function, the highest melatonin concentration (18 µg/mL) was associated with an increased heart rate compared to the negative control (*p* < 0.05; Fig. 2b). Lower concentrations either produced no significant changes or showed a tendency toward reduced heart rate, indicating a concentration-dependent effect on cardiac physiology.

### Behavioral Assessment

**Fig. 3:**
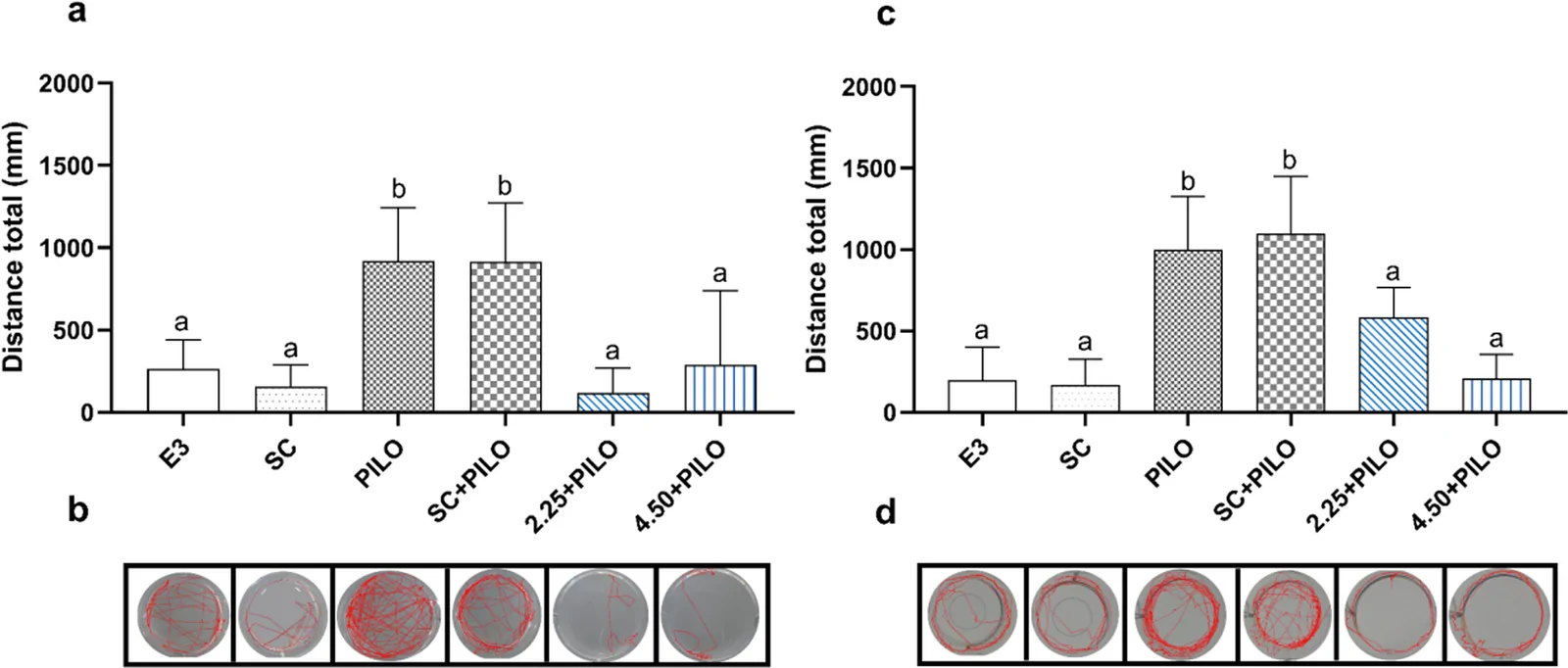
(**a**) Total distance traveled (mm) by 72 hpf *D. rerio* larvae pretreated with melatonin (2.25 and 4.50 µg/mL) and subsequently induced to epileptic seizures with 15 mM pilocarpine (PILO), including negative control (E3), solvent control (SC; 0.1% DMSO), solvent + PILO group (SC+PILO), and positive control (PILO); (**b**) Total distance traveled (mm) by 144 hpf *D. rerio* larvae exposed to the same experimental conditions. Data are presented as mean ± SD. Fifteen larvae per experimental group were analyzed at each developmental stage, with each larva considered one independent experimental unit (*n* = 15). Larvae evaluated at 72 and 144 hpf belonged to independent biological cohorts and were not repeatedly measured over time. Different letters indicate statistically significant differences between groups (*p* < 0.05). Data were analyzed using the Kruskal–Wallis test followed by Dunn’s post hoc test

At 72 hpf, larvae pretreated with melatonin (2.25 and 4.50 µg/mL) displayed significantly reduced locomotor activity following pilocarpine (PILO) exposure compared to the PILO and solvent + PILO groups (*p* < 0.05; Fig. 3a). Locomotor performance in these groups approached levels observed in the negative and solvent controls.

A similar pattern was observed at 144 hpf, where melatonin pretreatment attenuated PILO-induced hyperlocomotion (*p* < 0.05; Fig. 3b), indicating a consistent effect across developmental stages.

**Fig. 4:**
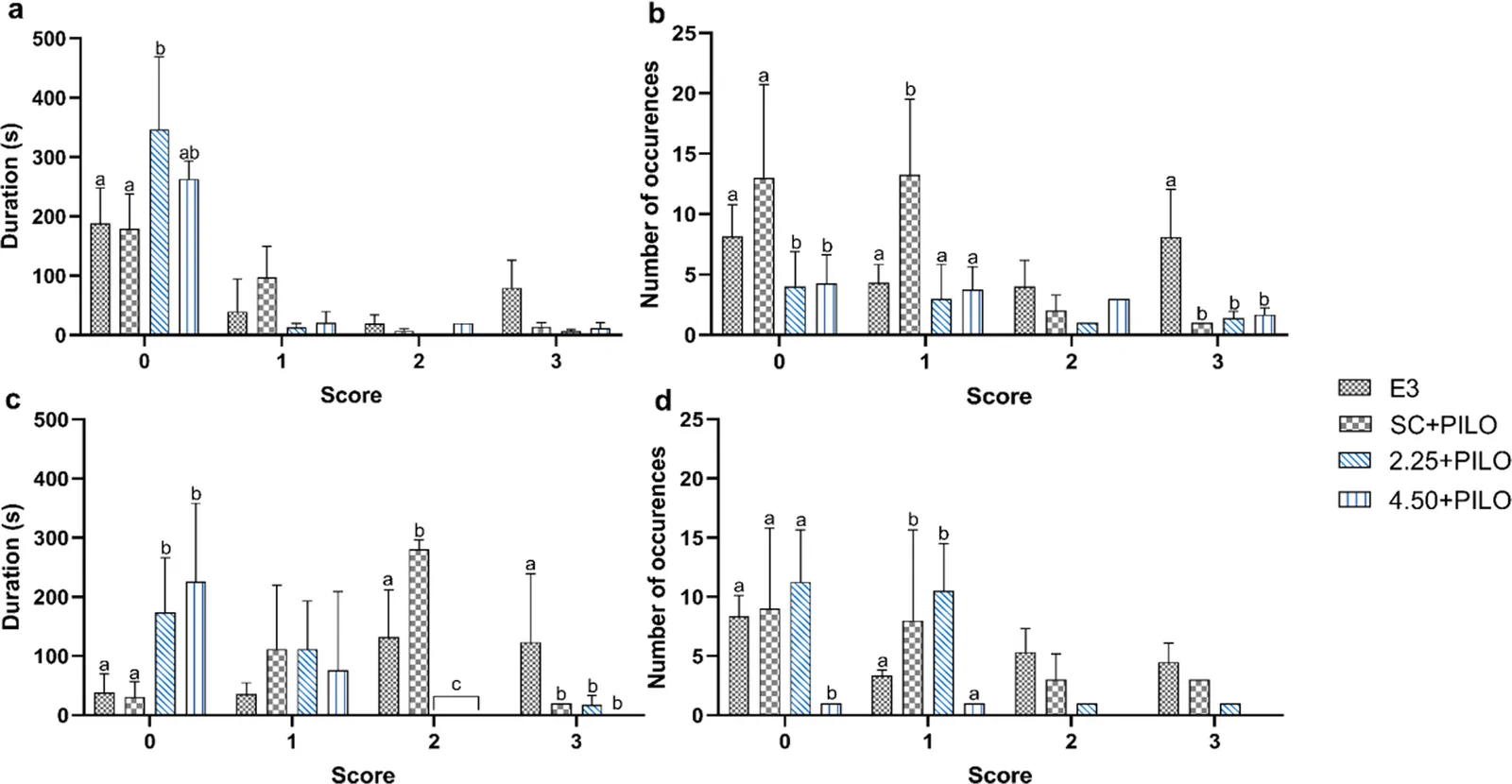
(**a**) Duration (s) of epileptic seizure scores in 72 hpf *D. rerio* larvae pretreated with melatonin (2.25–4.50 µg/mL) or solvent control (SC; 0.1% DMSO) and subsequently induced with 15 mM pilocarpine (PILO); (**b**) Number of occurrences of epileptic seizure scores in 72 hpf *D. rerio* larvae under the same experimental conditions; (**c**) Duration (s) of epileptic seizure scores in 144 hpf *D. rerio* larvae; (**d**) Number of occurrences of epileptic seizure scores in 144 hpf *D. rerio* larvae. Data are presented as mean ± SD. Fifteen larvae per experimental group were analyzed at each developmental stage, with each larva considered one independent experimental unit (*n* = 15). Larvae evaluated at 72 and 144 hpf belonged to independent biological cohorts and were not repeatedly measured over time. Different letters indicate statistically significant differences between groups (*p* < 0.05). Data were analyzed using the Kruskal–Wallis test followed by Dunn’s post hoc test

**Fig. 5:**
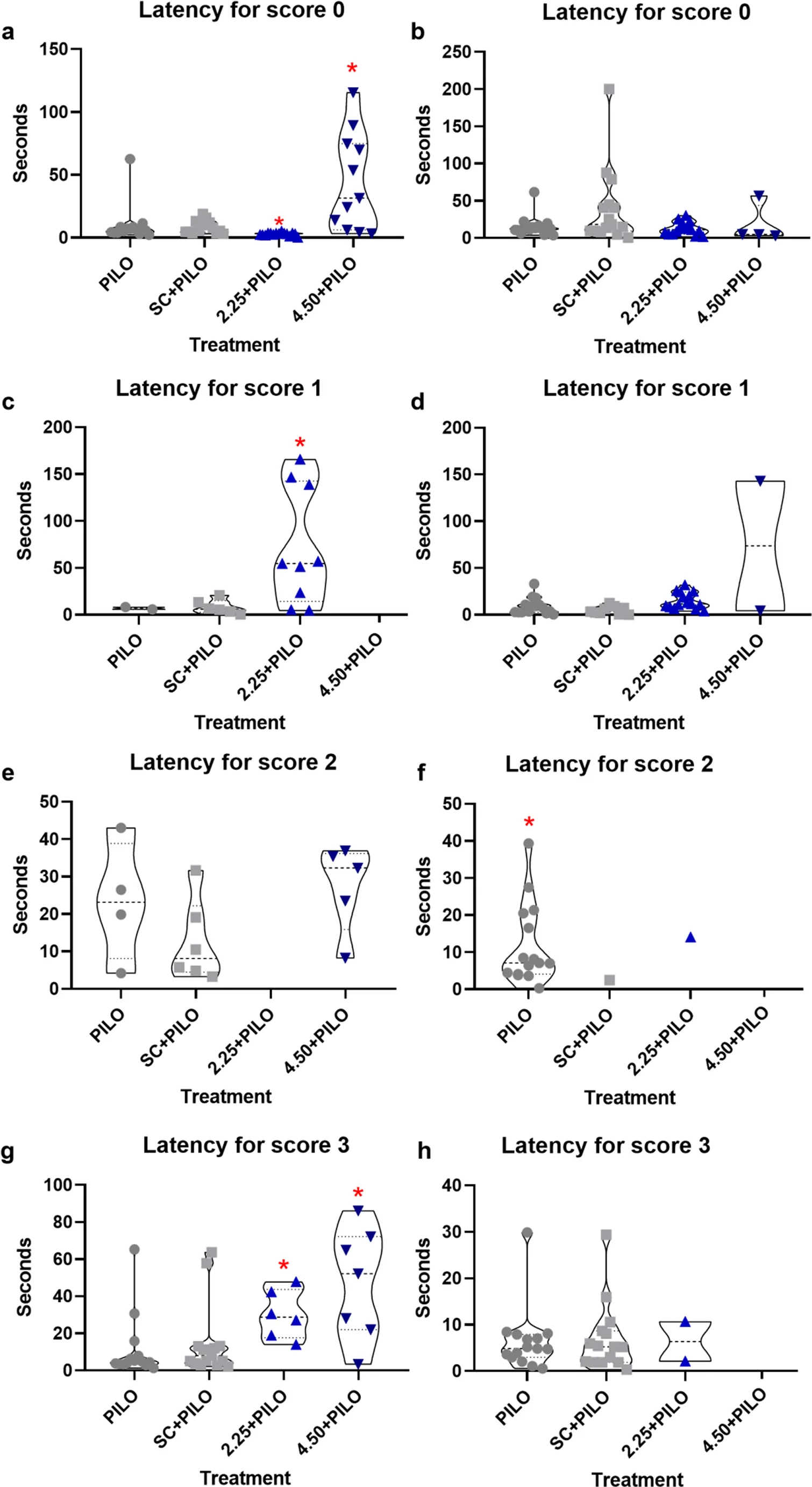
(**a**) Latency to the onset of score 0 in 72 hpf *D. rerio* larvae pretreated with melatonin (2.25 and 4.50 µg/mL) or solvent control (SC; 0.1% DMSO) and subsequently induced to epileptic seizures with 15 mM pilocarpine (PILO), including the positive control group (PILO); (**b**) Latency to the onset of score 0 in 144 hpf *D. rerio* larvae; (**c**) Latency to the onset of score 1 in 72 hpf *D. rerio* larvae; (**d**) Latency to the onset of score 1 in 144 hpf *D. rerio* larvae; (**e**) Latency to the onset of score 2 in 72 hpf *D. rerio* larvae; (**f**) Latency to the onset of score 2 in 144 hpf *D. rerio* larvae; (**g**) Latency to the onset of score 3 in 72 hpf *D. rerio* larvae; (**h**) Latency to the onset of score 3 in 144 hpf *D. rerio* larvae. Data are presented as mean ± SD. Fifteen larvae per experimental group were analyzed at each developmental stage, with each larva considered one independent experimental unit (*n* = 15). Larvae evaluated at 72 and 144 hpf belonged to independent biological cohorts and were not repeatedly measured over time. Symbols (*) indicate statistically significant differences between groups (*p* < 0.05). Data were analyzed using the Kruskal–Wallis test followed by Dunn’s post hoc test

In addition, melatonin significantly reduced seizure severity at both time points, as reflected by increased time spent in non-seizure behavior (score 0), shorter durations of seizure stages, and fewer seizure events (*p* < 0.0001; Fig. 4). Melatonin also increased latency to seizure onset and, at the higher concentration, limited progression to more severe seizure stages at 144 hpf (Fig. 5).

###  ROS Levels

**Fig. 6:**
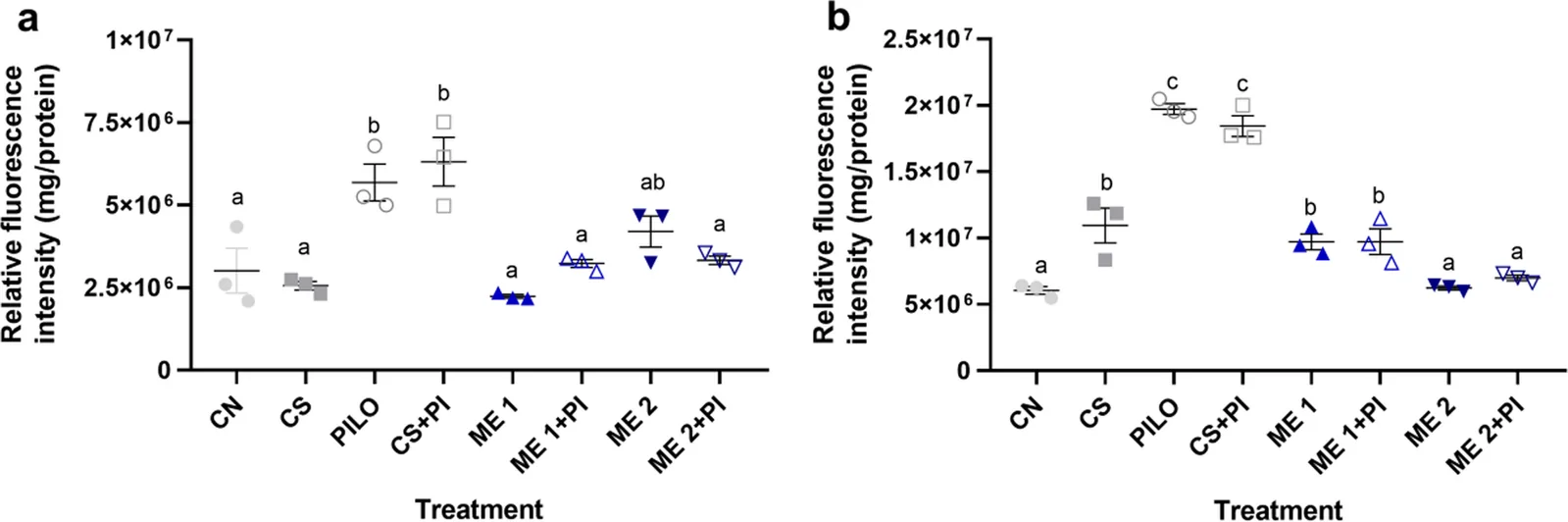
Relative fluorescence units (RFU) normalized to protein content in *D. rerio* larvae exposed to the following experimental conditions: negative control (E3), solvent control (SC; 0.1% DMSO), solvent + pilocarpine group (SC+PILO), positive control (PILO; 15 mM pilocarpine), melatonin 2.25 µg/mL + PILO, and melatonin 4.50 µg/mL + PILO. (**a**) ROS levels in larvae evaluated at 72 hpf; (**b**) ROS levels in larvae evaluated at 144 hpf. Data are presented as mean ± SD. Thirty larvae per experimental group were analyzed at each developmental stage, with each larva considered one independent experimental unit (*n* = 30). Larvae evaluated at 72 and 144 hpf belonged to independent biological cohorts and were not repeatedly measured over time. Different letters indicate statistically significant differences between groups (*p* < 0.05). Data were analyzed using the Kruskal–Wallis test followed by Dunn’s post hoc test

Melatonin pretreatment significantly reduced ROS levels in larvae exposed to PILO at both 72 and 144 hpf compared to the PILO and solvent + PILO groups (*p* < 0.05; Fig. 6). ROS levels in melatonin-treated groups were comparable to those observed in the negative control, indicating attenuation of PILO-associated oxidative stress. This reduction was consistent across both developmental stages.

## Discussion

The present study investigated the toxicological and behavioral effects of melatonin in a pilocarpine-induced seizure-like model using zebrafish larvae. Under the experimental conditions employed here, melatonin at 2.25 and 4.50 µg/mL was not associated with detectable developmental toxicity and was accompanied by reduced seizure-like behavioral responses and lower ROS levels under PILO-induced conditions at both 72 and 144 hpf.

The absence of significant effects on mortality and hatching agrees with previous studies reporting low embryotoxicity of melatonin in zebrafish and other experimental models (Danilova et al. 2004; Zhao et al. 2024). Nevertheless, the alterations observed at higher concentrations, particularly reduced spontaneous contractions and increased embryonic heart rate, indicate that melatonin exposure can induce concentration-dependent physiological disturbances during early development. These findings highlight the importance of dose selection when evaluating neuroactive compounds in developing organisms.

Under PILO-induced conditions, melatonin pretreatment attenuated hyperlocomotion, delayed seizure onset, and limited progression to more severe behavioral stages. Similar responses have been described in both zebrafish and mammalian seizure models, supporting the hypothesis that melatonin may modulate seizure-related processes during chemically induced seizure conditions (Costa-Lotufo et al. 2002; Scorza et al. 2009; Farias et al. 2022; Akyuz et al. 2021). In adult zebrafish exposed to kainic acid, melatonin reduced seizure intensity and oxidative stress markers, suggesting that these responses may extend across distinct seizure paradigms (Farias et al. 2022).

The present data are particularly relevant because cholinergic seizure models remain comparatively underexplored in zebrafish. Most available studies rely on PTZ-induced seizures, whereas pilocarpine activates neuronal pathways associated with muscarinic cholinergic signaling (D’Amora et al. 2023). In this regard, the current findings broaden the available evidence concerning dose-dependent melatonin responses in alternative seizure contexts.

Previous studies using mammalian pilocarpine models have produced heterogeneous results regarding melatonin efficacy. Some investigations reported reduced seizure occurrence and mortality following melatonin administration (Lima et al. 2006), whereas others failed to detect significant changes in seizure frequency or duration during chronic treatment protocols (Barateli et al. 2014). Collectively, these observations suggest that melatonin responses may depend on factors such as concentration, exposure duration, developmental stage, and experimental model.

The reduction in ROS levels observed under PILO-induced conditions is in line with previous reports linking melatonin to the modulation of oxidative balance during seizure-related conditions (Reiter et al. 2017; Ahmad et al. 2023; Farias et al. 2022). However, caution is warranted when interpreting these findings. Reduced oxidative stress may result from direct antioxidant actions, indirect consequences of lower neuronal hyperactivity, or the interaction of both processes (Lee et al. 2020). Because molecular and electrophysiological analyses were not performed, the present results do not allow mechanistic inferences regarding receptor signaling, neurotransmitter modulation, or mitochondrial regulation.

Likewise, although melatonin has previously been associated with GABAergic and glutamatergic modulation, the current findings support only behavioral and biochemical associations under seizure-like induction conditions (Farias et al. 2022). Thus, the observed responses should not be interpreted as direct evidence of anticonvulsant mechanisms but rather as indications of dose-dependent modulation of seizure-related outcomes.

The reduction in ROS levels observed under PILO-induced conditions is consistent with previous reports describing the antioxidant properties of melatonin during seizure-related conditions (Reiter et al. 2017; Ahmad et al. 2023; Farias et al. 2022). Oxidative stress is widely recognized as an important contributor to epileptogenesis and seizure-induced neuronal injury. Excessive ROS production promotes mitochondrial dysfunction, lipid peroxidation, protein oxidation, neuroinflammation, and neuronal hyperexcitability, thereby establishing a reciprocal relationship between oxidative damage and seizure activity (Aguiar et al. 2012; Pearson-Smith and Patel 2017; Kamieniak et al. 2024).

However, caution is warranted when interpreting these findings. Reduced oxidative stress may result from direct antioxidant actions of melatonin, indirect consequences of reduced neuronal hyperactivity, or the interaction of both mechanisms (Lee et al. 2020; Vasileva 2021). Because molecular, receptor-based, mitochondrial, and electrophysiological analyses were not performed, the present results do not allow mechanistic inferences regarding the pathways responsible for the observed behavioral improvement.

ROS levels were quantified using a DCFH-DA-based spectrofluorimetric assay performed on whole-larva homogenates. This quantitative biochemical approach has been widely applied in zebrafish studies investigating oxidative stress because it provides robust and reproducible measurements of total ROS production, allowing reliable comparisons among experimental groups (Mugoni et al. 2014; Ahmad et al. 2023; Farias et al. 2022). Although fluorescence microscopy could provide complementary information regarding the anatomical localization of ROS, particularly within the brain, it primarily offers spatial localization rather than quantitative assessment of global oxidative status. Since the objective of the present study was to quantitatively compare oxidative stress among experimental groups, the biochemical approach adopted here was considered appropriate for addressing the proposed hypothesis. Nevertheless, because whole-larva homogenates do not permit tissue-specific localization of oxidative damage, future studies combining quantitative biochemical analyses with fluorescence imaging or brain-specific oxidative stress markers will be valuable to determine whether the reduction in ROS occurs preferentially within neural tissues.

One limitation of the present study is the absence of melatonin-only groups in the behavioral seizure-like assessments. The experimental design was specifically structured to investigate whether melatonin could modulate behavioral alterations induced by pilocarpine exposure, rather than to characterize the isolated behavioral effects of melatonin under non-induced conditions. Therefore, the present findings should be interpreted within the context of PILO-induced responses.

Some limitations should be considered. Seizure-like activity was inferred exclusively from behavioral endpoints, without electrophysiological confirmation. Moreover, although larval zebrafish provide important advantages for rapid screening and developmental analyses, neuronal circuits continue to mature throughout larval development, which may influence seizure susceptibility and behavioral responses (Baraban et al. 2013). Consequently, this model does not fully reproduce the complexity of chronic epilepsy observed in mammalian systems. In addition, the mechanistic pathways discussed here were not directly investigated and should therefore be regarded as theoretical interpretations supported by previous literature rather than experimentally demonstrated mechanisms.

Taken together, the present findings indicate that melatonin produces concentration-dependent effects in zebrafish larvae exposed to pilocarpine. Lower concentrations were associated with attenuation of seizure-like behavioral responses and oxidative stress markers, whereas higher concentrations induced measurable physiological alterations. These results support further investigation of melatonin in cholinergic seizure models and reinforce the importance of careful dose selection in neurodevelopmental studies.

## Conclusion

Melatonin produced concentration-dependent effects in zebrafish larvae exposed to pilocarpine, with distinct behavioral and physiological outcomes across exposure levels. Lower concentrations were associated with reduced PILO-induced seizure-like behavioral responses and lower ROS levels, whereas higher concentrations were associated with measurable physiological changes during early development. The intensity of these responses also varied according to developmental stage, indicating that both exposure level and developmental stage influenced the observed outcomes.

Together, these findings contribute to the current evidence regarding melatonin responses in zebrafish seizure-like models and further support the use of larval zebrafish for investigating neuroactive compounds during early development. Although the present findings demonstrate consistent behavioral and biochemical responses, further studies integrating electrophysiological, molecular, and tissue-specific oxidative stress analyses will be necessary to elucidate the mechanisms underlying the neuroprotective effects of melatonin in cholinergic seizure models.

## Data Availability

No datasets were generated or analysed during the current study.
